# Phylogenetic Analysis of ALV-J Associated with Immune Responses in Yellow Chicken Flocks in South China

**DOI:** 10.1155/2021/6665871

**Published:** 2021-02-09

**Authors:** Qihong Zhang, Guodong Mo, Tingting Xie, Zihao Zhang, Huali Fu, Ping Wei, Xiquan Zhang

**Affiliations:** ^1^Guangdong Provincial Key Laboratory of Agro-Animal Genomics and Molecular Breeding, College of Animal Science, South China Agricultural University, Guangzhou 510642, China; ^2^Key Lab of Chicken Genetics, Breeding and Reproduction, Ministry of Agriculture, Guangzhou 510642, China; ^3^College of Animal Science and Technology, Guangxi University, Nanning 530004, China

## Abstract

The aim of this study was to better understand the sequence characteristics and immune responses in avian leukosis virus subgroup J (ALV-J) infected yellow chicken flocks in South China. We isolated four strains of ALV-J virus from these flocks, which were then identified by several methods, including subtype-specific polymerase chain reaction (PCR), enzyme-linked immunosorbent assay (ELISA), and immunofluorescence assay (IFA). All four viruses were sequenced for their complete genomes and named GD19GZ01, GD19GZ02, GD19GZ03, and GD19GZ04. In comparison with the reference sequence, the homology analysis showed that the *gag* and *pol* genes were relatively conserved, whereas *env* contained much variation. Both GD19GZ01 and GD19GZ02 almost entirely lacked the rTM region and E element, while the latter was retained in GD19GZ03 and GD19GZ04. Moreover, the virus replication levels in GD19GZ03 and GD19GZ04were much higher than those in GD19GZ01 and GD19GZ02. And three virus recombination events in GD19GZ01 and GD19GZ02 were revealed by the results of PDR5 and SimPlot software analysis. Additionally, we found that some interferon-stimulating genes (*CH25H*, *MX*, *PKR*, *OAS*, and *ZAP*) and inflammatory mediators (*IL-4*, *IL-6*, *IL-10*, *IL-12*, *1L-18*, and *TNF-α*) were significantly upregulated in the immune system organs of clinical chickens. Taken together, these findings clarify and reveal the sequence characteristics and trends in the variation of ALV-J infection in yellow chicken flocks of South China.

## 1. Introduction

Avian leukosis virus (ALV) is a type of avian retrovirus that belongs to the genus *Alpharetrovirus* of the Retroviridae family. According to the molecular biological characteristics of serum neutralization testing, host range, and envelope glycoproteins, the ALVs can be divided into 10 subgroups corresponding to A~J [1, 2]. The ALV-J is an exogenous virus, notable for its extremely high pathogenicity and infectivity, and it is far more harmful to chickens than are the other subgroups [3]. In China, the first record of poultry broilers found infected with ALV-J was in 1991. In recent years, the host range of ALV-J has expanded to the commercial egg layers and the breeds of China [4]. Studies have shown that ALV-J could induce different types of tumors in the liver, spleen, kidney, heart, bursa, thymus, and ovaries of chickens, such as hemangioma (He) and myeloma (ML) [5, 6]. Importantly, immunosuppression and immune tolerance are induced by ALV-J in infected birds, and this will significantly reduce the effectiveness of various vaccines, which could make birds more susceptible to secondary infections by other viruses or bacteria [7, 8]. Furthermore, ALV-J could coinfect host birds along with other ALV subgroups or even other viruses [10–13]. Due its RNA dimer structure and reverse transcriptase activity, as a retrovirus, ALV-J has a high frequency of mutation events, and it is prone to mutation or recombination.

The yellow chicken breeding industry is a crucial component of the larger poultry industry in South China. Yet, since many small farms still do not strictly screen and eradicate ALVs, coupled to the lack of effective vaccines or therapeutic drugs, the yellow chicken industry has been continuously threatened by ALV-J outbreaks in South China in recent years [9, 10]. In this study, four strains of the ALV-J virus were isolated from the egg-type yellow chicken at a local farm in South China. We cloned and sequenced the full-length ALV-J virus genomes and analyzed their molecular characteristics. Interestingly, whole-genome analysis of the virus sequence revealed the four strains belonged to the ALV J subgroup, but their molecular sequence characteristics and virus replication levels in vitro were significantly different. The results of this study enable a better understanding of the clinical symptoms and molecular characteristics of the virus sequence of ALV-J infections in chicken flocks of South China. This provides important timely data for clarifying the evolution and variation trends of ALV-J.

## 2. Materials and Methods

### 2.1. Sick Chicken Samples

A group of parental yellow chickens of the egg-type in a local farm in South China showed telltale symptoms of infection, such as a pale cockscomb, listlessness, a thin body, cluttered feathers, and obvious hemangiomas on their skin and digits. To understand the epidemiology ALVs' infection in yellow chickens, a total of 198 birds (40 weeks old) from that farm were analyzed. In short, anticoagulated blood samples were collected aseptically from each yellow chicken, whose blood was centrifuged to obtain plasma for the virus isolation. According to the previously described method [11], the single-layer DF-1 cells were infected with plasma, after which the cell supernatant was detected by an enzyme-linked immunosorbent assay (Avian Leukosis Virus Antibody Test Kit, IDEXX, USA). Positive individuals were dissected, and their tumor samples were collected aseptically, including the liver, spleen, and kidney, among others, which were fixed with 10% neutral formalin buffer or stored at −80°C till their later use.

### 2.2. Histopathology

Fresh tissues (liver, spleen, kidney) were collected, fixed with 10% neutral formalin buffer, then dehydrated, embedded in paraffin, sliced, and then stained with hematoxylin and eosin (HE) [12]. The prepared section samples were observed under a light microscope (ECLIPSE 80i Nikon, Japan) for histopathology.

### 2.3. Detection of ALVs

The peripheral blood was collected aseptically in an anticoagulation tube, of which 200 *μ*l of blood per chicken was taken for its DNA extraction, performed with the NRBC Blood DNA Kit (Omega, USA). This extracted blood DNA was used as the substrate for PCR. A previous methodology was referred to and followed to synthesize specific primers used for each subgroup of ALVs, which also included specific primers for the Marek's disease virus (MDV) and reticuloendothelial hyperplasia virus (REV) [13, 14]. The PCR reaction conditions were as follows: 95°C for 5 min, 95°C for 30 s, then 50°C to 60°C for 30 s, 72° for 45 s (32 cycles), ending with 72°C for 5 min (Table 1). The ensuing PCR products were detected on a 1% agarose gel (containing 0.5 *μ*g/ml ethylene bromide).

### 2.4. Virus Isolation

Since DF-1 cells were only sensitive to exogenous virus particles, the interference of endogenous virus replication could be ruled out [15]. The remaining anticoagulated blood in the preceding step (section above “Detection of ALVs”) was centrifuged at 1200 rpm and 4°C for 15 min. The resulting plasma was separated and stored at −80°C. According to a previously described method [9], the plasma samples were inoculated into a 24-well plate containing a DF-1 cell suspension (approximately 1.75 × 10^5^ cells/well), and the plate then placed at 37°C in the presence of 5% CO_2_. After 24 h, the culture solution was removed and replaced with fresh cell maintenance solution (1% Foetal Bovine Serum (FBS), 100 U/ml penicillin, and 100 U/ml streptomycin). These cells were maintained at 37°C under 5% CO_2_ for 5–7 days with daily monitoring. Supernatants and precipitated substrates were centrifuged and harvested after the cells had been frozen and thawed three times. According to the manufacturer's instructions, the supernatants were tested for ALV-specific antibodies (namely p27) by ELISA (IDEXX, USA). Then, according to the manufacturer's instructions, the precipitated substrates underwent DNA extraction, using the Tissue DNA Kit (Omega, USA), followed by the full-length amplification of the proviral genome by the PCR.

### 2.5. Proviral Genome Full-Length Amplification

According to the conserved region of the ALV-J original strain HPRS-103, four pairs of specific primers were designed [11], enabling the full-length viral genome to be amplified in segments. The gel-purified PCR product was ligated into a pMD18-T (Takara Biotechnology, Japan) cloning vector and transformed into DH5*α Escherichia coli* competent cells. After the transformation's completion, the bacterial solution was evenly spread on an agar plate containing ampicillin and incubated at 37°C for 12–16 h. Finally, the recombinant plasmid harboring the target DNA fragment detected by the PCR was sent to TSINGKE Biotech (Beijing, China) for sequencing. Three independent cloned plasmids were used in each group. The primer sequences are listed in Table 1.

### 2.6. Sequence Alignments and Analysis

All sequences were aligned and analyzed using DNAStar version 7.1. All the ALVs' strain reference sequences are from the National Center for Biotechnology Information (NCBI) GenBank database (their accession numbers can be found in Table 2). Phylogenetic analysis was performed using MEGA-X by applying neighbor-joining and maximum parsimony methods with 1000 bootstrap replicates [16]. The transcriptional regulatory elements of U3 in the ALV-J structure were analyzed using the online service system of NSITE (from Soft Berry).

### 2.7. Immunofluorescence Assay (IFA)

Those samples that tested positive for infection by ELISA were also tested by IFA. Briefly, the plasma was used to inoculate a monolayered 24-well plate. After incubation for 2 h, the supernatant was removed, and a 1% FBS cell maintenance solution was added to prolong the culture for 4–5 days. After fixing and sealing the cells, the ALV-specific monoclonal antibody JE9 (kindly provided by Dr. Aijian Qin, Yangzhou University, Yangzhou, China) as the primary antibody and FITC-labeled antimouse IgG (Bioss, Beijing, China) were used. Cell morphology was observed under a fluorescence microscope, with images recorded using NIS-Elements BR analysis software (Nikon, Japan).

### 2.8. RNA Isolation and qPCR

Total RNA was extracted using TRIzol reagent (Invitrogen) from tissues or cells and reversed-transcribed with a PrimeScript RT reagent kit with gDNA Eraser (Takara Biotechnology, Japan) according to the manufacturer's instructions. qPCR was performed on a Biorad CFX96 Real-Time Detection System using iTaq™ Universal SYBR® Green Supermix Kit reagents (Biorad, CA, USA). The 2^-*ΔΔ*Ct^ method was used to calculate relative expression changes. The primer sequences are listed in Table 3.

For the comparison experiment of virus replication in vitro, samples with a viral load of 10^4^TCID_50_/0.1 ml were used to inoculate DF-1 cells, and the supernatant and cells were collected at 1, 3, 5, and 7 dpi. The virus copy number was calculated by the absolute quantitative method.

### 2.9. Statistical Analyses

The data between two groups were analyzed by unpaired *t*-test if the data were in Gaussian distribution and had equal variance, or by unpaired *t*-test with Welch's correction if the data were in Gaussian distribution but with unequal variance, or by nonparametric test (Mann–Whitney *U* test) if the data were not normally distributed (Prism 8.0). Data were represented as means ± SD/SEM. Differences with*P* < 0.05were considered significant:^∗^*P* < 0.05,^∗∗^*P* < 0.01,^∗∗∗^*P* < 0.001. Differences within groups are marked with letters (A, a, B, b). The same letter means *P* < 0.05, between different uppercase or lowercase letters means *P* < 0.01, and between uppercase and lowercase letters means *P* < 0.001.

## 3. Results

### 3.1. Clinical Features and Histopathology of Sick Chickens

**Figure 1:**
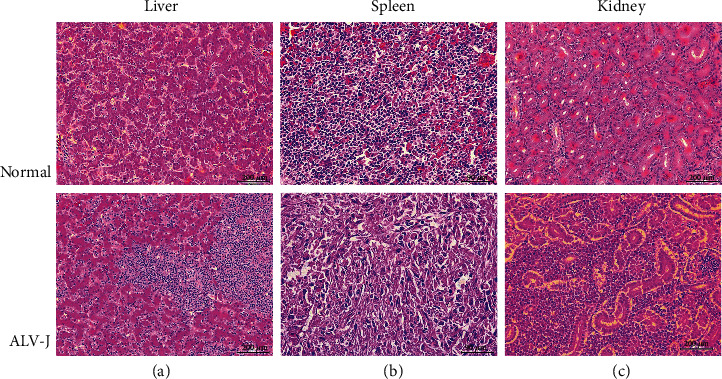
Histopathological changes in the clinical samples (hematoxylin and eosin staining). Comparisons between the normal group and ALV-J infection for (a) liver (bar = 200 *μ*M), (b) spleen (bar = 90 *μ*M), and (c) kidney (bar = 200 *μ*M) morphology of the chickens.

In this study, our experimental animals came from a group of egg-type chickens that were 40-week-old, which exhibited a pale cockscomb, bodyweight loss, mental depression, and decreased productivity. After detecting the ALVs' specificity antibody p27, it was found that a total of 15.7% (31/198) of the sampled chickens were positive for infection and the mortality rate was 3.5% (7/198). In particular, we found that the joints and diameter of the toes of these sick chickens were swollen, with blood blisters and ruptures evident, and signs of bleeding and hemangioma on their skin (Supplementary Figure 1A-1C). The pathological anatomy analysis revealed that the liver was abnormally swollen and almost occupied the entire abdominal cavity, with diffuse tumor nodules of different sizes on the liver surface that were either spherical or flat in shape. Also observed was a diffuse, bright red flower speckled texture as well as a solid texture on the liver. Moreover, there were also sick chickens whose liver edges appeared off-white, with mottled off-white nodules visible in the cross-section, characterized by a soft texture. Other internal organs also betrayed varying degrees of disease (Supplementary Figure 1D-1F). Histopathologically, in normal liver tissues, the morphology of healthy liver lobules could be distinguished. But after the ALV-J infection, many small round tumor cells infiltrated the liver lobules in the liver tissue of the sickened chickens (Figure 1(a)). Normal spleen tissue was alternately composed of red pulp and white pulp, yet AVL-J infection damaged the structure of the spleen and widened the intercellular space (Figure 1(b)). Similarly, numerous inflammatory cells infiltrated the kidney, which squeezed and deformed this organ's tubules (Figure 1(c)).

### 3.2. Identification and Isolation of Virus

**Figure 2:**
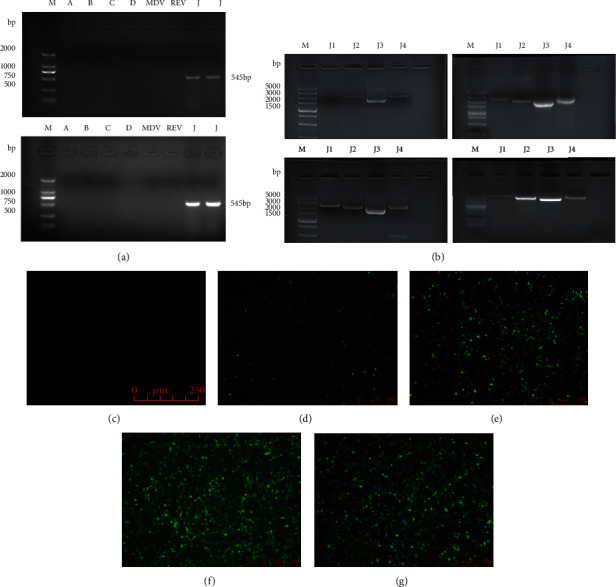
Identification of the four virus strains by PCR reaction and IFA response. In panel (a) are the PCR products that were electrophoresed on an agarose gel, and the four viruses at 545 bp, showing the specific bands. (b) Agarose electrophoresis results of the full-length ALV-J genome amplified from the four strains of viruses. (c) Uninfected DF-1 cells as a negative control, at 100×. (d–g) GD19GZ01 through GD19GZ04 strains inoculated into the DF-1 cells, respectively; the IFA response indicating specific green fluorescence, at 100×. The numbers in the upper column of the gel chart are for the following: M is the marker, A to D are the ALV-A- to ALV-D-specific primers, J is the ALV-J specific primers for H5/H7, and J1 to J4 represent the four segments of the full length of the ALV-J genome. bp: base pairs. The numbers on the left indicate the lengths of the molecular weight standards.

Among the 31 positive chickens, we selected the four samples with the highest ELISA value for virus identification and isolation (s/p value of p27). We synthesized specific primers for different subgroups of ALV, which also included those for MDV and REV. The four samples tested positive when using the ALV-J specific primers H5/H7, with a specific band evident at 545 bp, while the other subgroup primers had negative results (Figure 2(a)). Similarly, the ALV-J specific monoclonal antibody JE9 was used for the IFA experiments; this showed positive results as revealed by the strong green fluorescence (Figures 2(c)–2(f)). Therefore, the virus carried by these four samples was ALV-J. We then performed a full-length amplification of the provirus genome and successfully isolated four strains of ALV-J (Figure 2(b)), here named GD19GZ01 through GD19GZ04, respectively. Among them, the fluorescence intensity of GD19GZ03 and GD19GZ04 was higher than that of GD19GZ01 and GD19GZ02 (Figures 2(d)–2(g)).

### 3.3. Sequence Analysis Results of the Four Isolates Compared with Reference Strains

After alignment with the sequence characteristics of the original ALV-J strain HPRS-103 (GenBank accession number: Z46390), the full-length proviral genomes of GD19GZ01 to GD19GZ04 were obtained, which were 7479–7626 nucleotides (nt) in size, respectively. They all had the classic structure of a retrovirus, featuring the Long Terminal Repeated- (LTR-) leader-*gag*-*pol*-*env*-LTR, but they did not contain a viral oncogene, thus indicating that the four isolates belonged to the chronic tumor-causing avian leukemia virus. The four isolates shared 85.1%–98.0% nucleotide identity when compared with the reference strains. The detailed results of these pairwise comparisons are presented in Table 4.

**Figure 3:**
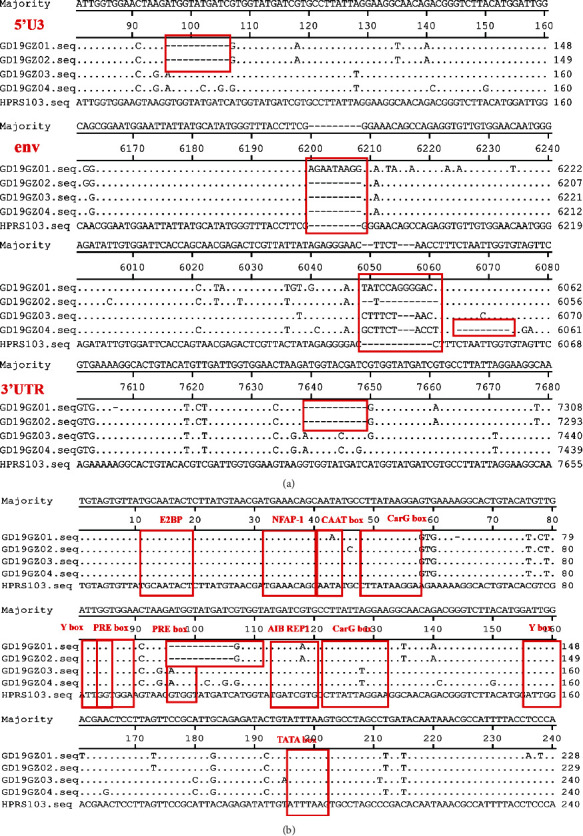
Sequence analysis results of the proviral genome. (a) The four isolates have different degrees of insertions and deletions in their 5′U3, *env*, and 3′UTR regions when compared with those of the original strain HPRS-103. (b) Transcriptional regulatory elements in the 5′U3 region of the four isolates. The dots (.) indicate identical residues, while the letters indicate the base substitutions. The dashes (-) indicate gaps in the alignment. Locations of deletions or insertions are boxed and marked.

Compared with the classic strain HPRS-103, the main reason for the difference in length between the four isolates was the redundant nonfunctional TM (rTM) region and downstream E element. Both GD19GZ01 and GD19GZ02 lacked the rTM region and E element almost entirely; however, GD19GZ03 and GD19GZ04 lacked the rTM region but retained the E element (Supplementary Figure 2A-2B). Notably, GD19GZ01 and GD19GZ02 each had 11-bp deletions in their 5′U3 and 3′UTR regions (between the bases 95–107 and 613–7625, following the HPRS-103 numbering scheme, same below). GD19GZ01 also had 9 bp insertion on the *env* gene (between bases 6187-6188). The four isolates had different degrees of insertions and deletions in their 5′U3, *env*, and 3′UTR regions, when compared with the original strain HPRS103 (Figure 3(a)).

In terms of the coding region, when compared to the reference strains, the *gag* and *pol* genes of the four strains were relatively conserved, sharing a 94.5%–99.1% nucleotide identity and 95.7%–99.6% amino acid identity, respectively. Yet the *env* gene, which could encode envelope proteins, was the most variable; with it, the four isolates shared only a 56.1%–97.0% nucleotide identity and a 47.1%–95.2% amino acid identity, being able to encode 575, 570, 571, and 568 amino acids in the *env* gene, respectively (Table 4).

The transcriptional regulatory elements of the four isolates in the U3 region were relatively conserved, namely with respect to 1 CAAT, E2BP, NFAP-1, AIB REP1, TATA Box, 2 CarG, Y, and PRE Box. Due to the 43rd base mutation (T/A), the GD19GZ01 virus strain had lost its CAAT Box (Figure 3(b)).

### 3.4. Phylogenetic Analysis of the Four Isolates

**Figure 4:**
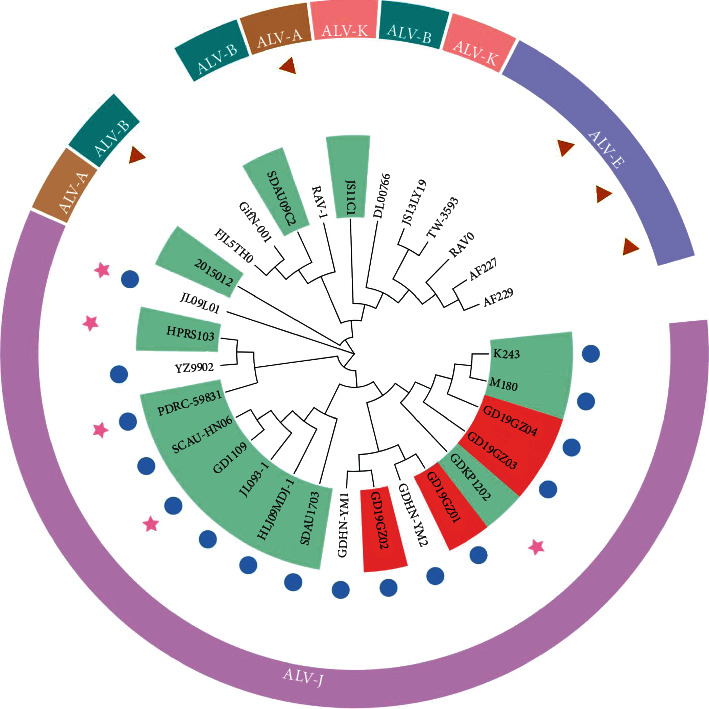
Phylogenetic relationships of the four isolates to the reference strains, based on the proviral genome sequence. The red background indicates the four isolates and the green background indicates the reference strains that retained the E element. The blue circle indicates hemangioma (He), pink stars indicate myeloma (ML), brown triangles indicate lymphoma leukosis (LL).

The phylogenetic analysis of the 26 reference sequences and four isolates revealed that the latter formed the same cluster as other isolates in South China in recent years. In this respect, GD19GZ01 through GD19GZ04 have the highest consistency with GDHN-YM2, GDHN-YM1, K243, and M180, respectively. There were also obvious regional differences among the same subgroups, and the sequence identity of strains from the same region was relatively high (Figure 4).

### 3.5. All Positions of the Three Recombinants Were between the Gag and Pol Genes

**Figure 5:**
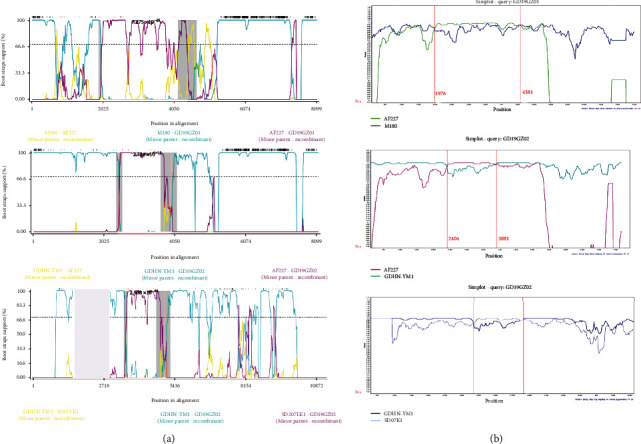
Determination of reorganization events. (a) The PDR5 analyzed the recombination events of the virus isolates. The bootscan was based on the pairwise distance model, with a window size of 200, a step size of 50, and 1000 bootstrap replicates generated by the RDP5. (b) The SimPlot analysis graph of reorganized breakpoints. These were based on a 200-bp window; each step is 20 bp for comparison (100 self-expanding, setting a 90% consistent tree, neighbor-joining method, Kimura two-parameter model).

Several potential recombination events were detected by PDR5, but only three of them were accepted as being robust. The recombination sequence of event 1 was for GD19GZ01, and its parental sequences were M180 (ALV-J) and AF227 (ALV-E); that of event 2 was for GD19GZ02 and its parental sequences were GDHN-YM1 (ALV-J) and AF227 (ALV-E), and that of event 3 was GD19GZ02 and its parental sequences were GDHN-YM1 (ALV-J) and SD07LK1 (ALV-J) (Figures 5(a) and 5(b)). All positions of the three recombinants were between the *gag* and *pol* genes. The *P* values for the six algorithm results (from PDR5 software) are in Supplementary Table 1.

### 3.6. Differential Expression of Innate Immune Genes and Inflammatory Cytokines in Different Organs of the ALV-J Infection

**Figure 6:**
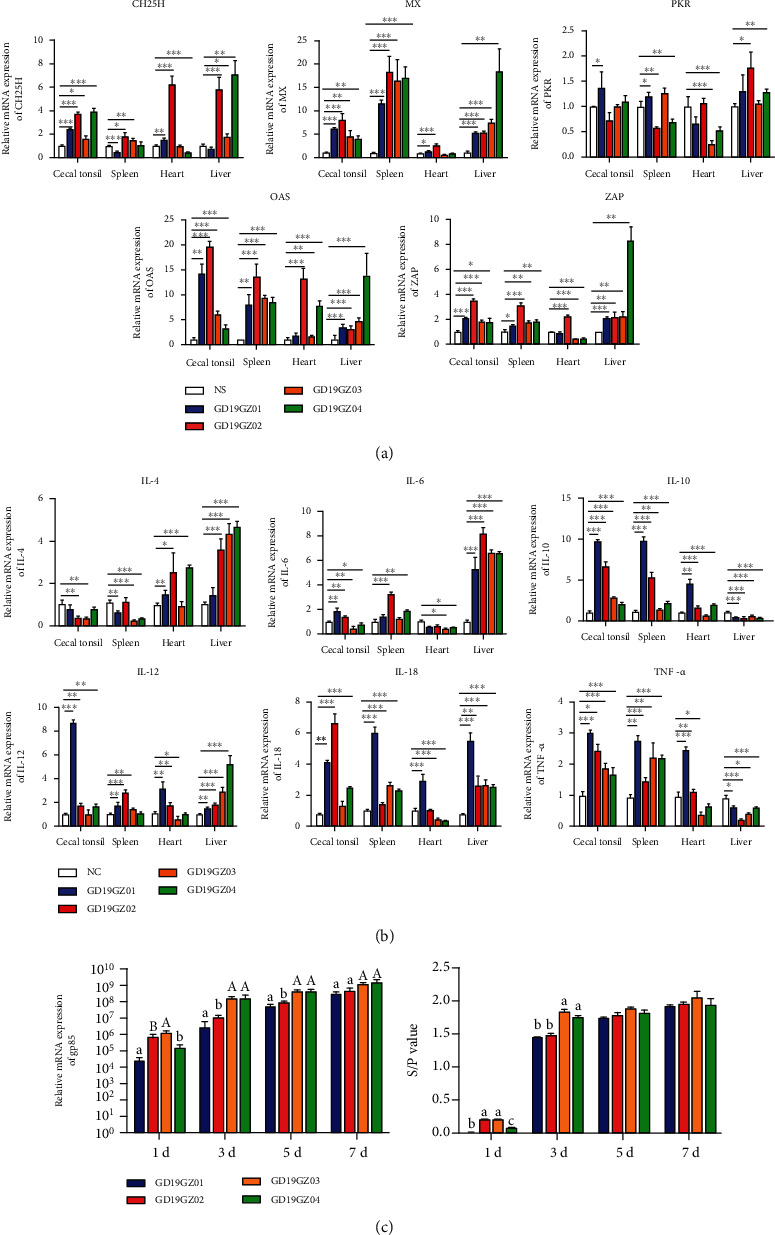
Expression of the innate immune genes and inflammatory cytokines in different organs of sick chickens and comparison of virus replication in vitro. The expression levels (a) of ISGs and (b) proinflammatory cytokines and anti-inflammatory cytokines in the immune organs. (c) Viral synthesis levels of the four isolates, as determined by qPCR and ELISA assays (the s/p value indicates the viral load). The data shown are the means ± SEM. The differences with *P* < 0.05 were considered significant. ^∗^*P* < 0.05, ^∗∗^*P* < 0.01, ^∗∗∗^*P* < 0.001 (a, b). Differences within groups are marked with letters (A, a, B, b). The same letter means *P* < 0.05, between different uppercase or lowercase letters means *P* < 0.01, and between uppercase and lowercase letters means *P* < 0.001 (c).

Interferon resists virus invasion by inducing the production of interferon-stimulating genes (ISGs), and this is an important line of defense for the innate immune response [17]. These ISGs, such as *CH25H*, *MX*, *PKR*, *OAS*, and *ZAP*, have been proven to block virus invasion in different ways to protect cells from infection [18–22]. Therefore, we analyzed the transcriptional levels of related ISGs in different immune organs in the sick chickens. The gene expression for *CH25H*, *MX*, *OAS*, and *ZAP* was significantly upregulated in the cecal tonsils, kidneys, and livers of the four sick chickens (Figure 6(a)). At the same time, we further explored the expression levels of inflammatory cytokines in the immune organs of the same four sick chickens. In their cecal tonsils, *IL-4* and *IL-6* show a trend of downward regulation, whereas the *IL-10* and proinflammatory cytokines *IL-12*, *IL-18*, and *TNF-α* were significantly upregulated. In the spleen, except for *IL-4*, the other five inflammatory cytokines were significantly upregulated. In the liver, the genes for *IL-4*, *IL6*, *IL12*, and *IL-18* were differentially expressed and upregulated, while both *IL-10* and *TNF-α* were downregulated (Figure 6(b)).

The DF-1 cells were infected with a viral load of 10^4^TCID_50_/0.1 ml, after which the level of viral RNA synthesis and viral particle content were detected at different time points. The qPCR results indicated that GD19GZ02 and GD19GZ03 underwent higher levels of replication at 1 dpi (days postinoculation) than the other two strains, a difference that was an extremely significant difference. However, the virus replication levels of GD19GZ03 and GD19GZ04 became the highest at 3, 5, and 7 dpi. The results of ELISA and qPCR were basically the same. It is worth noting that GD19GZ04 had a low level of virus replication early on, at 1 dpi, but later on, the virus was clearly capable of a higher level of replication. At any time period, the virus replication level of GD19GZ01 was the lowest among the four isolates (Figure 6(c)).

## 4. Discussion

Since ALVs were included in the “National animal disease prevention and control for the medium and long term planning,” through strict selection measures, the prevention and control of ALVs have made great progress [23]. Although ALVs have been completely eradicated in other countries, there are still reports of ALVs' outbreak in China among scattered farms and a variety of chicken species [24, 25]. The yellow chicken breeding industry, a pivotal part of the poultry industry in South China, has been continuously attacked by ALV-J in recent years. Similarly, the yellow feather broiler breeders have faced grave problems due to ALV-J infections [26]. Therefore, it is necessary to learn more of the pathogenesis and molecular sequence characteristics of ALV-J infection in yellow chicken flocks in South China.

In this study, four strains of ALV-J were isolated from the flocks of yellow chickens in South China, and their similarity, phylogenetic analysis, and sequence features were evaluated. GD19GZ01 and GD19GZ02 almost completely lacked the rTM region and E element region, while GD19GZ03 and GD19GZ04 each retained their E element. The four isolates were isolated from the same flock of yellow chickens, but their structural characteristics differed significantly, further highlighting the complexity of the ALV infection in these chicken flocks.

Among the 26 virus reference sequences that we selected, 50% (13/26) of those virus isolates featured an E element deletion. Coincidentally, the number of strains lacking the rTM region was also 50%, so whether it was an isolate that caused either hemangioma (He) or myeloma (ML), the rTM region might still be lost. This suggests that losing the rTM region was probably not related to the type of tumor elicited in the host. Nevertheless, only 15.3% (4/26) of the isolates simultaneously lacked both the rTM region and E element; evidently, then, the deletion of these two regions at once is a relatively rare phenomenon. The specific function of the E element remains unclear, however. Some researchers believe that despite not being essential for tumorigenesis induction by viruses, the E element nonetheless contributes to oncogenicity in certain genetic lines of chicken [27]. In the comparison with virus replication levels in vitro, we found that GD19GZ02, GD19GZ03, and GD19GZ04 were under the same conditions of the transcriptional regulatory elements in the U3 region, but the in vitro replication levels of GD19GZ03 and GD19GZ04, which retained the E element, were significantly higher than those of GD19GZ01 and GD19GZ02 that lacked it. So, for GD19GZ01, its loss of a transcriptional regulatory element (CAAT box) might explain why it had the lowest level of viral replication at any time. Unfortunately, we have not verified in vivo whether strains that retain the E element are more tumorigenic.

The unique pathogenicity of ALV-J is considered to be closely related to its special *env* gene, which encodes two proteins, GP85 and GP37, of which the former is highly variable while the latter is relatively conserved [28, 29]. In our study, the *env* gene's variation in the four isolates was relatively large, and its consistency with the other reference strains was only 56.1%–97%. Most of these mutations occurred in the hypervariable region hr1, hr2, and the variable region vr3 of GP85.

Chicken type-I interferons (I-IFN) play a dominant role in the chicken immune system and mediate the first line of defense against viral pathogens infecting the avian species [30]. When IFNs bind to their specific receptors, a cascade of signals is activated that results in the induction of several ISGs, thus establishing the antiviral state in the infected cells [31]. Therefore, we analyzed the expression levels of some ISGs in the immune organs of four sick chickens. These results indicated that *CH25H*, *MX*, *OAS*, and *ZAP* were all significantly upregulated in multiple immune organs (Figure 6(a)). Among them, *CH25H* was proven to inhibit the replication of ALV-J by producing 25HC [18]. However, the immunogenic features of most ISGs are still unknown. Exploring the antiviral potential of ISGs is recommended, since they could serve as valuable targets for therapeutic use against ALV-J and also as potential candidates for vaccine development; this is one of several strategies to deal with ALV-J.

A dynamic and ever-shifting equilibrium exists between proinflammatory cytokines and anti-inflammatory components of the immune system [30]. Accordingly, to investigate deviations from that, we analyzed the expression levels of cytokines in the immune organs of four sick chickens under long-term invasion by ALV-J. The anti-inflammatory cytokines *IL-4* and *IL-6* were significantly upregulated in the liver, and both are known to have marked inhibitory effects on the expression and release of proinflammatory cytokines [32]. Therefore, the release of *TNF-α* was inhibited in the liver. Although *IL-10* was downregulated in the liver of chickens, it was significantly upregulated in the cecal tonsils, spleen, and heart (Figure 6(b)). The invasion of ALV-J caused a strong inflammatory response in the immune organs of yellow chickens, as evinced by proinflammatory cytokines *IL-12*, *IL-18*, and *TNF-α* that were all significantly upregulated in most of their immune organs. It is worth mentioning that the expression of anti-inflammatory cytokines and proinflammatory cytokines increased abnormally in GD19GZ01. Despite complexities inherent in inflammatory cytokines, therapeutic interventions with specific cytokine inhibitors or anti-inflammatory cytokines have already been shown to confer significant clinical benefits in humans [33]. However, our current knowledge of the immune response caused by ALV-J invading yellow chickens is incomplete, so further research on this aspect is needed.

Finally, we used software to detect the occurrence of three viral recombination events on GD19GZ01 and GD19GZ02, all of which were found between the *gag* and *pol* genes. In recombination events 1 and 2, the recombination fragment is composed of an exogenous retrovirus and an endogenous retrovirus. Actually, in the early days, there was a view that ALV-J is a virus obtained by mutual recombination of exogenous retrovirus and endogenous retrovirus [34]. It was worth noting that of all 143 isolates in the NCBI database (updated August 1, 2020), a total of 316 recombination events were detected. ALVs' recombination not only occurs between different subgroups but also between ALVs and other viruses [35, 36]. The occurrence of recombination events means that viruses continue to evolve under selective pressure, and the final result may be the emergence of new subgroups.

In summary, we isolated four ALV-J from yellow chickens on a farm in South China and performed detailed sequence analyses of them. The results further demonstrate the complexity of ALVS infection in chicken flocks in different regions. Our findings thus help to reveal the clinical symptoms and sequence characteristics of ALV-J' infection in yellow chickens in South China and, more generally, they provide valuable data for elucidating the evolution and variation trends of ALVs.

## Figures and Tables

**Table 1:** The primers used to detect the ALVs and to amplify the full-length proviral genome of ALV-J isolates.

Primer^a^	Sequence (5′–3′)	Annealing temperature (°C)	Size of PCR products (bp)
MDV-F	GGATGAGGTGACTAAGAAAG	59	560
MDV-R	CGAACCAAAGGTAACACACG		
REV-F	GCCTTAGCCGCCATTGTA	53	383
REV-R	CCAGCCTACACCACGAACA		
ALV-A-F	GGATGAGGTGACTAAGAAAG	50	692
ALV-A-R	AGAGAAAGAGGGGTGTCTAAGGAG		
ALV-B-F	GGATGAGGTGACTAAGAAAG	50	847
ALV-B-R	TGGACCAATTCTGACTCATT		
ALV-C-F	GGATGAGGTGACTAAGAAAG	50	860
ALV-C-F	GAGGCCAGTACCTCCCACG		
ALV-D-F	GGATGAGGTGACTAAGAAAG	50	797
ALV-D-F	ATCCATACGCACCACAGTATTCG		
ALV-J-H5	GGATGAGGTGACTAAGAAAG	55	545
ALV-J-H7	CGAACCAAAGGTAACACACG		
F: J-1F	TGTAGTGTTATGCAATACTCTT	52	2100
F: J-1R	CGACCCAGTTTGTCCATCCCTC		
F: J-2F	AGCACCCTCCACGCTGACCAC	59	2100
F: J-2R	GGTGGTCGGTAACCCTCACTTTCA		
F: J-3F	GACCCTGCCCTGCCTCTGGAA	59	1800
F: J-3R	CGACGGAAATAATAACCACGCACA		
F: J-4F	GCGTGGTTATTATTTCCGTCGTCC	59	2400
F: J-4R	TGAAGCCTTCCGCTTCATGCAGGT		

**Table 2:** The ALV-J reference strains used in this study.

ALV strains	Country	Year	Host	Tumor phenotype	Subgroup	Accession number
RAV-1	USA	1980	Commercial chicken	LL	A	MF926337
2015012	China	2015	N/A	N/A	A	KY612442
SDAU09C2	China	2009	N/A	N/A	B	HM446005
FJ15TH0	China	2016	Hetian chicken	LL	B	KX839489
DL00766	China	2018	Hy-line variety brown	N/A	B & E	MH454773
RAV0	USA	1980	Commercial layer chicken	LL	E	MF817822
AF227	USA	2010	Commercial broiler	LL	E	MF817820
AF229	USA	2010	Commercial broiler chicken	LL	E	MF817821
JS11C1	China	2012	Chinese indigenous chicken	N/A	K	KF746200
JS13LY19	China	2018	Chicken	N/A	K	MG770235
HPRS-103	UK	1989	Parental meat-type chicken	ML	J	Z46390
SCAU-HN06	China	2007	Commercial layer chicken	He	J	HQ900844
PDRC-59831	USA	2007	Broiler	He and ML	J	KP284572
TW-3593	China Taiwan	2008	Taiwan country chickens	N/A	N/A	HM582658
HLJ09MDJ-1	China	2009	Layer chicken	He	J	JN624880
JL093-1	China	2009	Layer hens	He	J	JN624878
JL09L01	China	2009	Commercial egg-type chickens	He and ML	J	HQ148555
YZ9902	China	2010	White meat-type chicken	He	J	HM235670
GD1109	China	2011	Commercial layer chicken	He and ML	J	JX254901
GDKP1202	China	2012	Local commercial broilers	ML	J	JX453210
GifN_001	Japan	2015	N/A	N/A	N/A	MK757486
M180	China	2016	Late-feathering Chinese yellow chicken	N/A	J	KX611834
K243	China	2016	Fast-feathering Chinese yellow chicken	N/A	J	KX611833
GDHN-YM1	China	2018	Commercial layer chicken	He	J	MK683478
GDHN-YM2	China	2018	Commercial layer chicken	He	J	MK683479
SDAU1703	China	2018	Hy-line chicken	He	J	KY980659

ML: myeloid leukosis; He: hemangioma; LL: lymphoid leukosis; N/A: data not available.

**Table 3:** The primers used for the qPCR.

Primer^a^	Sequence (5′–3′)	Accession no.
*GAPDH-F*	GAACATCATCCCAGCGTCCA	NM_204305.1
*GAPDH-R*	CGGCAGGTCAGGTCAACAAC	
*CH25H-F*	AATCCAGCCGCAGAGCTATC	NM_001277354.1
*CH25H-R*	CAGCTCTGGAGCTATCACCG	
*OSA-F*	CAGCGCCTGTACACCGAG	NM_205041.1
*OSA-R*	GGTTCTCCAGCTCCTTGGTC	
*PKR-F*	CCTCTGCTGGCCTTACTGTCA	NM_204487.1
*PKR-R*	AAGAGAGGCAGAAGGAATAATTTGCC	
*ZAP-F*	TTGATTCGGCGCCTCTCTAC	NM_001012938.1
*ZAP-R*	ACTGGCCGTGGTCATTCTTC	
*MX-F*	TTGTCTGGTGTTGCTCTTCCT	NM_204609.1
*MX-R*	GCTGTATTTCTGTGTTGCGGTA	

**Table 4:** The nucleotide and amino acid identity shared between GD19GZ01 and the other reference strains.

ALV strains	Nucleotide sequences (%)					Amino acid sequences (%)		
	Whole genome	LTR	*Gag*	*Pol*	*Env*	*Gag*	*Pol*	*Env*
GD19GZ02	97.8	93.7	98.1	98.8	96.0	97.7	99.4	92.6
GD19GZ03	95.8	93.0	96.3	97.3	94.0	97.4	98.4	91.4
GD19GZ04	96.3	97.4	96.5	98.0	95.3	97.4	98.6	92.2
RAV-1	85.6	86.5	96.2	97.1	56.5	97.0	97.7	47.8
2015012	86.8	93.9	95.2	97.1	57.4	96.7	98.3	48.2
SDAU09C2	86.0	95.0	96.6	96.8	57.2	97.7	98.6	48.8
FJ15TH0	86.0	90.7	95.3	97.7	57.6	97.0	98.5	48.8
DL00766	85.1	65.7	96.2	98.5	56.7	97.6	99.0	47.2
RAV0	85.1	63.5	96.7	98.3	56.5	97.4	98.7	48.3
AF227	85.2	64.6	96.6	98.5	56.6	97.9	98.7	48.3
AF229	85.2	64.6	96.7	98.5	56,6	97.9	98.7	48.1
JS11C1	85.2	79.7	94.6	97.9	57.7	96.2	98.4	47.9
JS13LY19	85.3	63.8	96.7	98.3	57.2	98.0	98.6	47.6
HPRS-103	94.7	92.7	96.0	97.3	93.7	96.7	98.6	90.5
SCAU-HN06	95.2	89.5	95.3	97.8	93.7	96.9	99.0	91.9
PDRC-59831	94.7	90.4	94.9	96.8	93.3	96.2	98.3	88.9
TW-3593	85.2	66.1	96.4	98.1	57.0	97.6	98.7	47.6
HLJ09MDJ-1	95.4	86.9	96.3	97.4	95.0	96.7	98.5	92.0
JL093-1	95.4	87.2	96.3	97.5	94.6	96.9	98.5	92.4
JL09L01	94.6	90.4	96.8	97.1	90.0	97.4	97.5	87.7
YZ9902	94.3	92.3	95.0	97.0	93.7	95.9	98.4	89.4
GD1109	95.3	90.4	95.3	98.0	94.9	97.0	98.7	92.3
GDKP1202	95.9	93.0	96.6	96.8	95.1	98.1	98.9	92.8
GifN_001	85.6	89.9	95.3	97.7	56.7	97.0	98.5	49.5
M180	96.2	94.6	96.5	97.4	95.1	97.6	98.9	91.7
K243	95.9	94.2	96.2	97.4	94.0	97.2	98.7	90.4
GDHN-YM1	97.1	97.4	97.4	97.8	95.5	97.3	99.0	93.5
GDHN-YM2	98.0	99.0	97.5	98.7	96.8	97.6	99.3	93.7
SDAU1703	94.9	89.5	96.3	97.3	93.0	96.9	98.5	90.5

## Data Availability

The data supporting the results of our study can be found within this manuscript.

## Supplementary Materials

Supplementary Materials Supplementary Table 1. The *P* values of the isolate recombination events are based on the six algorithms of PDR. At least five algorithms with *P* values <1 × 10^−10^ were required to accept a robust event. Supplementary Figure 1. Clinical symptoms of sick chicken infected with ALV-J. (A) Swollen toe joints and bleeding. (B) Blood blisters present in the diameter joints. (C) Hemangiomas on the skin. (D) An abnormally enlarged liver almost filling the entire abdominal cavity. (E) A liver cross-section showing grayish white nodules. (F) Kidney swollen with gray-white nodules. Supplementary Figure 2. Comparison of the four isolates to the original strain HPRS-103 with respect to their rTM and E element regions. (A) The four isolates completely lacked a redundant nonfunctional TM (rTM) region. (B) GD19GZ01 and GD19GZ02 almost completely lacked the E element, while GD19GZ03 and GD19GZ04 retained it. The dots (.) indicate identical residues, while the letters indicate base substitutions. The dashes (-) indicate gaps in the alignment. Locations of deletions or insertions are boxed and marked.

## References

[1] Barnard R. J. O., Elleder D., Young J. A. T. (2006). Avian sarcoma and leukosis virus-receptor interactions: from classical genetics to novel insights into virus-cell membrane fusion. *Virology*.

[2] Payne L. N., Gillespie A. M., Howes K. (1992). Myeloid leukaemogenicity and transmission of the HPRS-103 strain of avian leukosis virus. *Leukemia*.

[3] Gao Y., Qin L., Pan W. (2010). Avian leukosis virus subgroup J in layer chickens, China. *Emerging Infectious Diseases*.

[4] Sun S., Cui Z. (2007). Epidemiological and pathological studies of subgroup J avian leukosis virus infections in Chinese local "yellow" chickens. *Avian Pathology Journal of the W.v.p.a*.

[5] Abolnik C., Wandrag D. B. (2014). Avian gyrovirus 2 and avirulent Newcastle disease virus coinfection in a chicken flock with neurologic symptoms and high mortalities. *Avian Diseases*.

[6] Feng M., Dai M., Xie T., Li Z., Shi M., Zhang X. (2016). Innate immune responses in ALV-J infected chicks and chickens with hemangioma in vivo. *Frontiers in Microbiology*.

[7] He S., Zheng G., Zhou D., Li G., Cheng Z. (2019). Clonal anergy of CD117+chB6+ B cell progenitors induced by avian leukosis virus subgroup J is associated with immunological tolerance. *Retrovirology*.

[8] Wang P., Lin L., Shi M. (2020). Vertical transmission of ALV from ALV-J positive parents caused severe immunosuppression and significantly reduced marek's disease vaccine efficacy in three-yellow chickens. *Veterinary Microbiology*.

[9] Min F., Yan T., Dai M. (2016). Endogenous retrovirus ev21 dose not recombine with ALV-J and induces the expression of ISGs in the host. *Frontiers in Cellular & Infection Microbiology*.

[10] Qi S., Yang L., Li W. (2018). Molecular characteristics of avian leukosis viruses isolated from indigenous chicken breeds in China. *Poultry Science*.

[11] Li Y., Liu X., Liu H. (2013). Isolation, identification, and phylogenetic analysis of two avian leukosis virus subgroup J strains associated with hemangioma and myeloid leukosis. *Veterinary Microbiology*.

[12] CHENG Z., LIU J., CUI Z., ZHANG L. (2010). Tumors associated with avian leukosis virus subgroup J in layer hens during 2007 to 2009 in China. *The Journal of Veterinary Medical Science*.

[13] Lai H., Zhang H., Ning Z. (2011). Isolation and characterization of emerging subgroup J avian leukosis virus associated with hemangioma in egg-type chickens. *Veterinary Microbiology*.

[14] Wang P., Shi M., He C. (2019). A novel recombinant avian leukosis virus isolated from gamecocks induced pathogenicity in Three-Yellow chickens: a potential infection source of avian leukosis virus to the commercial chickens. *Poultry science*.

[15] Maas R., Zoelen D. V., Oei H., Claassen I. (2006). Replacement of primary chicken embryonic fibroblasts (CEF) by the DF-1 cell line for detection of avian leucosis viruses. *Biologicals*.

[16] Pan W., Gao Y., Qin L. (2012). Genetic diversity and phylogenetic analysis of glycoprotein GP85 of ALV-J isolates from Mainland China between 1999 and 2010: Coexistence of two extremely different subgroups in layers: coexistence of two extremely different subgroups in layers. *Veterinary Microbiology*.

[17] Goraya M. U., Zaighum F., Sajjad N., Anjum F. R., Rahman S. U. (2019). Web of interferon stimulated antiviral factors to control the influenza A viruses replication. *Microbial pathogenesis*.

[18] Xie T., Feng M., Dai M., Mo G., Zhang X. (2019). Cholesterol-25-hydroxylase is a chicken ISG that restricts ALV-J infection by producing 25-hydroxycholesterol. *Viruses*.

[19] Mpenda F. N., Keambou C. T., Kyallo M., Pelle R., Buza J. (2019). Polymorphisms of the chicken mx gene promoter and association with chicken embryos’ susceptibility to virulent newcastle disease virus challenge. *BioMed Research International*.

[20] Schulz O., Pichlmair A., Rehwinkel J. (2010). Protein kinase R contributes to immunity against specific viruses by regulating interferon mRNA integrity. *Cell Host & Microbe*.

[21] Silverman R. H. (2007). Viral encounters with 2′,5′-oligoadenylate synthetase and RNase L during the interferon antiviral response. *Journal of Virology*.

[22] Zhu Y., Chen G., Lv F. (2011). Zinc-finger antiviral protein inhibits HIV-1 infection by selectively targeting multiply spliced viral mRNAs for degradation. *Proceedings of the National Academy of ences of the United States of America*.

[23] Meng F., Li Q., Zhang Y., Cui Z., Chang S., Zhao P. (2018). Isolation and characterization of subgroup J Avian Leukosis virus associated with hemangioma in commercial Hy-Line chickens. *Poultry science*.

[24] Zhang Q., Zhao D., Guo H., Cui Z. (2010). Isolation and identification of a subgroup A avian leukosis virus from imported meat-type grand-parent chickens. *Virologica Sinica*.

[25] Zhao D. M., Zhang Q. C., Cui Z. Z. (2010). Isolation and identification of a subgroup B avian leukosis virus from chickens of Chinese native breed Luhua. *Bing Du Xue Bao*.

[26] Bo W., Qing-yuan L. I., Shao-qiong L., Yong-guang Z., Zhi-zhong C., Sun S. H. (2011). Evaluation on ALV Infection in Fertilized Eggs from A Wan-nan Yellow-feather Parent Broiler Breeder Flock. *Chinese Journal of Animal and Veterinary Sciences*.

[27] Chesters P. M., Smith L. P., Nair V. (2006). E (XSR) element contributes to the oncogenicity of Avian leukosis virus (subgroup J). *The Journal of General Virology*.

[28] Chesters P. M., Howes K., Petherbridge L., Evans S., Payne L. N., Venugopal K. (2002). The viral envelope is a major determinant for the induction of lymphoid and myeloid tumours by avian leukosis virus subgroups A and J, respectively. *The Journal of General Virology*.

[29] Jiang L., Zeng X., Hua Y. (2014). Genetic diversity and phylogenetic analysis of glycoprotein gp85 of avian leukosis virus subgroup J wild-bird isolates from Northeast China. *Archives of Virology*.

[30] Opal S. M., DePalo V. A. (2000). Anti-Inflammatory Cytokines. *CHEST*.

[31] Keestra A. M., de Zoete M. R., Bouwman L. I., Vaezirad M. M., van Putten J. P. (2013). Unique features of chicken Toll-like receptors. *Developmental and Comparative Immunology*.

[32] Brown M. A., Hural J. (1997). Functions of IL-4 and control of its expression. *Critical Reviews in Immunology*.

[33] Tepler I., Elias L., Smith J. W., Hussein M. N., Kaye J. A. (1996). A randomized placebo-controlled trial of recombinant human interleukin-11 in cancer patients with severe thrombocytopenia due to chemotherapy. *Blood*.

[34] Smith L. M., Toye A. A., Howes K., Bumstead N., Payne L. N., Venugopal K. (1999). Novel endogenous retroviral sequences in the chicken genome closely related to HPRS-103 (subgroup J) avian leukosis virus. *The Journal of General Virology*.

[35] Cui Z., Sun S., Zhang Z., Meng S. (2009). Simultaneous endemic infections with subgroup J avian leukosis virus and reticuloendotheliosis virus in commercial and local breeds of chickens. *Avian Pathology*.

[36] Wang P., Yang Y., Lin L., Li H., Wei P. (2017). Complete genome sequencing and characterization revealed a recombinant subgroup B isolate of avian leukosis virus with a subgroup J-like U3 region. *Virus Genes*.

