# Supplementary material for: Phylogenetic Analysis of ALV-J Associated with Immune Responses in Yellow Chicken Flocks in South China
Source: Mediators Inflamm. 2021 Feb 9;2021:6665871. doi: 10.1155/2021/6665871 (PMC7886527; doi:10.1155/2021/6665871)
Supplement: Supplementary Materials — Supplementary Table 1. The P values of the isolate recombination events are based on the six algorithms of PDR. At least five algorithms with P values <1 × 10^−10^ were required to accept a robust event. Supplementary Figure 1. Clinical symptoms of sick chicken infected with ALV-J. (A) Swollen toe joints and bleeding. (B) Blood blisters present in the diameter joints. (C) Hemangiomas on the skin. (D) An abnormally enlarged liver almost filling the entire abdominal cavity. (E) A liver cross-section showing grayish white nodules. (F) Kidney swollen with gray-white nodules. Supplementary Figure 2. Comparison of the four isolates to the original strain HPRS-103 with respect to their rTM and E element regions. (A) The four isolates completely lacked a redundant nonfunctional TM (rTM) region. (B) GD19GZ01 and GD19GZ02 almost completely lacked the E element, while GD19GZ03 and GD19GZ04 retained it. The dots (.) indicate identical residues, while the letters indicate base substitutions. The dashes (-) indicate gaps in the alignment. Locations of deletions or insertions are boxed and marked. [file 6665871.f1.zip › Supplementary Description_6665871.docx]

Supplementary Description:

**Supplementary Table 1. The P-values of the isolate recombination events are based on the six algorithms of PDR.** At least five algorithms with P-values < 1×10^-10^ were required to accept a robust event

**Supplementary Figure 1. Clinical symptoms of sick chicken infected with ALV-J.**

(A) Swollen toe joints and bleeding. (B) Blood blisters present in the diameter joints. (C) Hemangiomas on the skin. (D) An abnormally enlarged liver almost filling the entire abdominal cavity. (E) A liver cross section showing grayish white nodules. (F) Kidney swollen with gray-white nodules.

**Supplementary Figure 2. Comparison of the four isolates to the original strain HPRS-103 with respect to their rTM and E element regions.**

(A) The four isolates completely lacked a redundant non-functional TM (rTM) region. (B) GD19GZ01 and GD19GZ02 almost completely lacked the E element, while GD19GZ03 and GD19GZ04 retained it. The Dots (.) indicate identical residues, while the letters indicate base substitutions. The dashes (-) indicate gaps in the alignment. Locations of deletions or insertions are boxed and marked
