# Supplementary material for: Phylogenetic Analysis of ALV-J Associated with Immune Responses in Yellow Chicken Flocks in South China
Source: Mediators Inflamm. 2021 Feb 9;2021:6665871. doi: 10.1155/2021/6665871 (PMC7886527; doi:10.1155/2021/6665871)
Supplement: Supplementary Materials — Supplementary Table 1. The P values of the isolate recombination events are based on the six algorithms of PDR. At least five algorithms with P values <1 × 10^−10^ were required to accept a robust event. Supplementary Figure 1. Clinical symptoms of sick chicken infected with ALV-J. (A) Swollen toe joints and bleeding. (B) Blood blisters present in the diameter joints. (C) Hemangiomas on the skin. (D) An abnormally enlarged liver almost filling the entire abdominal cavity. (E) A liver cross-section showing grayish white nodules. (F) Kidney swollen with gray-white nodules. Supplementary Figure 2. Comparison of the four isolates to the original strain HPRS-103 with respect to their rTM and E element regions. (A) The four isolates completely lacked a redundant nonfunctional TM (rTM) region. (B) GD19GZ01 and GD19GZ02 almost completely lacked the E element, while GD19GZ03 and GD19GZ04 retained it. The dots (.) indicate identical residues, while the letters indicate base substitutions. The dashes (-) indicate gaps in the alignment. Locations of deletions or insertions are boxed and marked. [file 6665871.f1.zip › Supplementary materials.docx]

**SUPPLEMENTARY MATERIALS**

**Supplementary Table**

| Events | Recombinants | Major parent | Minor parent | Methods | | | | | |
| --- | --- | --- | --- | --- | --- | --- | --- | --- | --- |
|  |  |  |  | RDP | GENECONV | BootScan | MaxChi | Chimaera | Siscan |
| 1 | GD19GZ01 | M180 | AF227 | 2.466×10^-48^ | 1.031×10^-34^ | 4.213×10^-35^ | 1.588×10^-12^ | 1.860×10^-03^ | 5.751×10^-15^ |
| 2 | GD19GZ02 | GDHN-YM1 | AF227 | 4.351×10^-46^ | 1.579×10^-42^ | 4.4651×10^-45^ | 1.877×10^-10^ | 2.314×10^-10^ | - |
| 3 |  | GDHN-YM1 | SD07LK1 | 2.053×10^-16^ | 1.312×10^-11^ | 2.724×10^-14^ | 1.106×10^-07^ | 7.052×10^-10^ | 3.214×10^-10^ |

**Supplementary Table 1. The P-values of the isolate recombination events are based on the six algorithms of PDR.** At least five algorithms with P-values < 1×10^-10^ were required to accept a robust event.


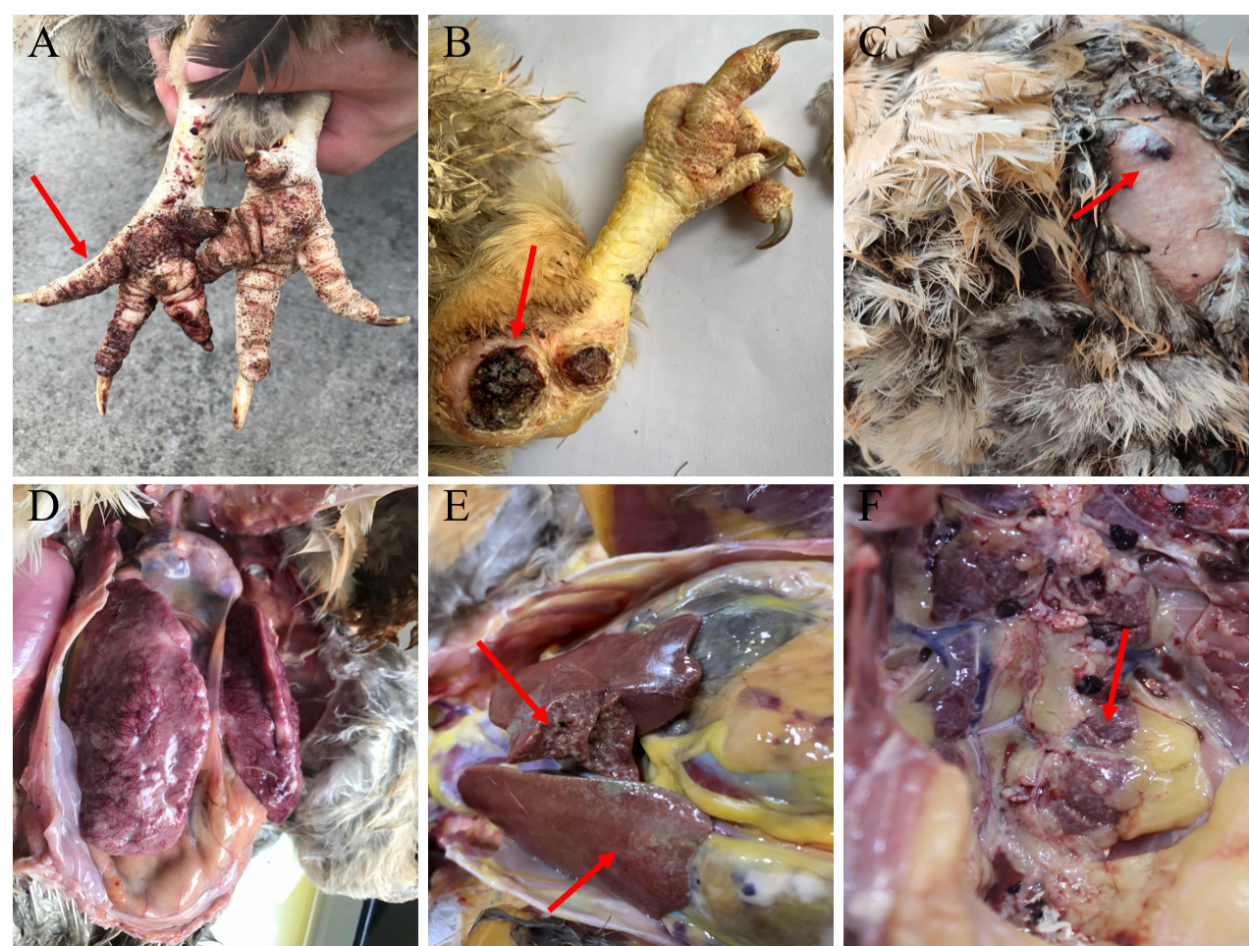


**Supplementary Figure 1. Clinical symptoms of sick chicken infected with ALV-J.**

(A) Swollen toe joints and bleeding. (B) Blood blisters present in the diameter joints. (C) Hemangiomas on the skin. (D) An abnormally enlarged liver almost filling the entire abdominal cavity. (E) A liver cross section showing grayish white nodules. (F) Kidney swollen with gray-white nodules.


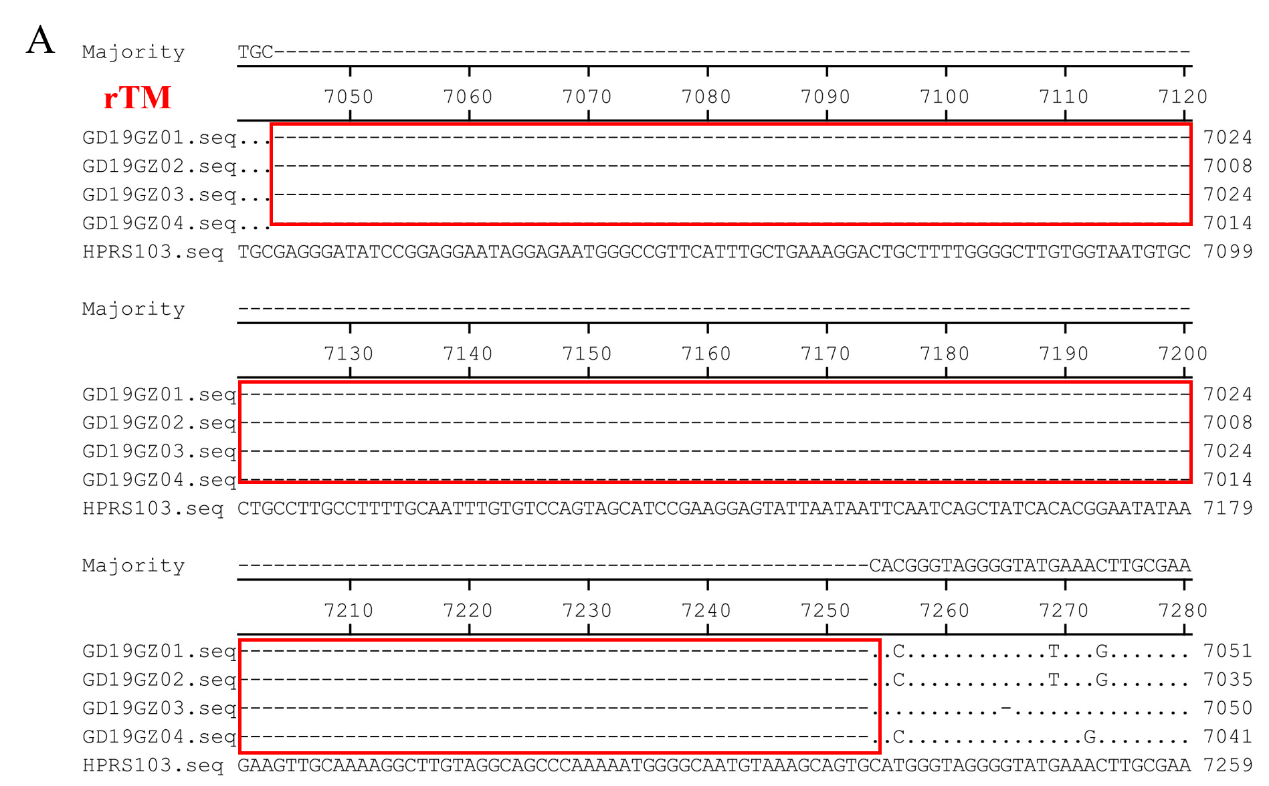


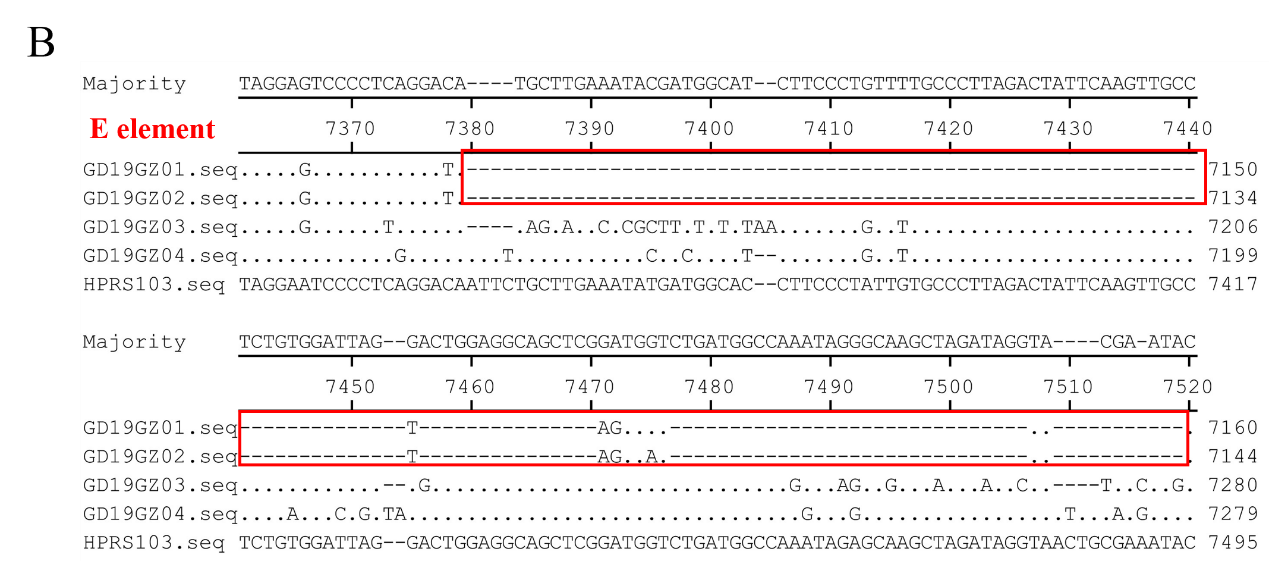


**Supplementary Figure 2. Comparison of the four isolates to the original strain HPRS-103 with respect to their rTM and E element regions.**

(A) The four isolates completely lacked a redundant non-functional TM (rTM) region. (B) GD19GZ01 and GD19GZ02 almost completely lacked the E element, while GD19GZ03 and GD19GZ04 retained it. The Dots (.) indicate identical residues, while the letters indicate base substitutions. The dashes (-) indicate gaps in the alignment. Locations of deletions or insertions are boxed and marked.
